# Effect of resistance training on physical function during chemotherapy in colon cancer

**DOI:** 10.1093/jncics/pkae058

**Published:** 2024-07-16

**Authors:** Justin C Brown, Shengping Yang, Stephanie L E Compton, Kristin L Campbell, Elizabeth M Cespedes Feliciano, Sara Quinney, Barbara Sternfeld, Bette J Caan, Jeffrey A Meyerhardt, Kathryn H Schmitz

**Affiliations:** Pennington Biomedical Research Center, Baton Rouge, LA, USA; Louisiana State University Health Sciences Center New Orleans School of Medicine, New Orleans, LA, USA; Stanley S. Scott Cancer Center, Louisiana State University Health Sciences Center, New Orleans, LA, USA; Pennington Biomedical Research Center, Baton Rouge, LA, USA; Pennington Biomedical Research Center, Baton Rouge, LA, USA; University of British Columbia, Vancouver, BC, Canada; Kaiser Permanente Northern California, Oakland, CA, USA; Indiana University School of Medicine, Indianapolis, IN, USA; Kaiser Permanente Northern California, Oakland, CA, USA; Indiana University School of Medicine, Indianapolis, IN, USA; Dana-Farber Cancer Institute, Boston, MA, USA; University of Pittsburgh, Pittsburgh, PA, USA

## Abstract

**Background:**

The decline of physical function during chemotherapy predicts poor quality of life and premature death. It is unknown if resistance training prevents physical function decline during chemotherapy in colon cancer survivors.

**Methods:**

This multicenter trial randomly assigned 181 colon cancer survivors receiving postoperative chemotherapy to home-based resistance training or usual care control. Physical function outcomes included the short physical performance battery, isometric handgrip strength, and the physical function subscale of the Medical Outcomes Short-Form 36-item questionnaire. Mixed models for repeated measures quantified estimated treatment differences.

**Results:**

At baseline, participants had a mean (SD) age of 55.2 (12.8) years; 67 (37%) were 60 years or older, and 29 (16%) had a composite short physical performance battery score of no more than 9. Compared with usual care control, resistance training did not improve the composite short physical performance battery score (estimated treatment difference = −0.01, 95% confidence interval [CI] = −0.32 to 0.31; *P* = .98) or the short physical performance battery scores for balance (estimated treatment difference = 0.01, 95% CI = −0.10 to 0.11; *P* = .93), gait speed (estimated treatment difference = 0.08, 95% CI = −0.06 to 0.22; *P* = .28), and sit-to-stand (estimated treatment difference = −0.08, 95% CI = −0.29 to 0.13; *P* = .46). Compared with usual care control, resistance training did not improve isometric handgrip strength (estimated treatment difference = 1.50 kg, 95% CI = −1.06 to 4.05; *P* = .25) or self-reported physical function (estimated treatment difference = −3.55, 95% CI = −10.03 to 2.94); *P* = .28). The baseline short physical performance battery balance score (*r *=* *0.21, 95% CI = 0.07 to 0.35) and handgrip strength (*r *=* *0.23, 95% CI = 0.09 to 0.36) correlated with chemotherapy relative dose intensity.

**Conclusion:**

Among colon cancer survivors with relatively high physical functioning, random assignment to home-based resistance training did not prevent physical function decline during chemotherapy.

**Clinical Trial Registration:**

NCT03291951.

Cancer survivors experience accelerated declines in physical function compared with cancer-free controls (1,2). Physical function is a key determinant of quality of life and predicts overall survival in cancer survivors (3,4). Poor functional status is associated with an increased risk of severe chemotherapy toxicity (5). Muscle mass and strength are associated with physical function among older adults without cancer (6). Therefore, the decline in physical function in cancer survivors may occur partly from treatment-induced muscle strength and mass impairments (7,8). In cancer survivors, physical functioning may be a biomarker that quantifies the performance and coordination of various physiologic systems susceptible to treatment-induced decline (9).

These observations provided the scientific rationale to examine physical function as an endpoint in a randomized controlled trial of resistance training vs usual care control during postoperative chemotherapy for colon cancer (10). We previously reported that random assignment to resistance training did not statistically significantly improve the coprimary endpoints of chemotherapy relative dose intensity and patient-reported chemotherapy toxicities compared with usual care control (11). This report describes the physical function outcomes. We hypothesized that random assignment to resistance training would prevent physical function decline during chemotherapy compared with usual care control.

## Methods

### Study design

The study used a randomly assigned, parallel-group, controlled design conducted at 3 US centers (Kaiser Permanente Northern California, Oakland, CA; Dana-Farber Cancer Institute, Boston, MA; and Penn State Cancer Institute, Hershey, PA). The study followed good clinical practice and the ethical principles in the Declaration of Helsinki. The institutional review board at each study center approved the protocol and informed consent document. All participants provided informed consent and approval from their physician before completing any study activities. The study was registered on Clinicaltrials.gov as NCT03291951. The trial design is described elsewhere (10).

### Subjects

Eligible  participants were adults (aged 18 years and older) with histologically proven stage II-III colon cancer who had completed curative-intent tumor resection and planned to initiate postoperative chemotherapy. Participants had no additional surgery planned within the intervention period (including colostomy reversal) and no contraindications to physical activity as determined by the physical activity readiness questionnaire (12). Eligible participants did not engage in resistance training more than twice weekly in the previous 3 months and did not have metastatic cancer or untreated hypertension. Details of the eligibility criteria are described elsewhere (10).

### Random assignment and blinding

Participants were randomly assigned in a 1:1 ratio to resistance training or usual care control. Participants were stratified by sex (male vs female), cancer stage (II vs III), planned chemotherapy duration (3 months vs 6 months), planned chemotherapy regimen (FOLFOX vs CAPOX vs capecitabine monotherapy vs 5-fluorouracil and leucovorin), and study center (Kaiser Permanente Northern California vs Dana-Farber Cancer Institute vs Penn State Cancer Institute). Group assignment for each participant was determined with a minimization procedure, which uses adaptive stratified sampling to balance the specified stratification factors (13). Outcome assessors were blinded to treatment assignment, but participants were not blinded to treatment assignment.

### Usual care control group

Participants assigned to the usual care control group were advised to ask their physician what exercise would be safe and effective. No specific guidance about exercise was provided.

### Resistance training group

Participants assigned to the resistance training group performed home-based weight-lifting exercises. The progressive resistance training program was consistent with recommendations from the American College of Sports Medicine (ACSM) (14,15). Participants received 4-6 individualized training sessions with a certified cancer exercise trainer to learn how to complete each exercise; after the training sessions, exercises were unsupervised. Because of the SARS-CoV-2 outbreak (March 2020), these in-person sessions were converted to virtual sessions for the remainder of the study. Resistance exercises used adjustable dumbbell weights (PowerBlock Inc, Burnsville, MN, USA) provided by the study. The program included 5 large muscle exercises (eg, bench press, squats, 1-arm row, lunges, and deadlifts) performed in 3-5 sets of 6-10 repetitions at an intensity of 65%-85% of the 1-repetition maximum twice weekly for the duration of chemotherapy. A rest of 60-120 seconds between sets was encouraged. The initial starting weight for each exercise was established with a submaximal strength test for each exercise to estimate the 1-repetition maximum strength (16). The study provided each participant with 40 grams daily of whey protein (Agropur Inc, Eden Praire, MN, USA) to maximize the anabolic response to resistance training (17).

### Physical function outcome measures

Outcome measures were obtained before, but no later than, 6 weeks of starting chemotherapy (baseline; before random assignment) and within 4 weeks after completing chemotherapy (end of study). The short physical performance battery is a brief functional assessment based on a balance test, timed short-distance walk, and repeated chair stands (18,19). For standing balance, participants were asked to maintain balance for a maximum of 10 seconds in each of 3 positions, characterized by a progressive narrowing of the base support: side-by-side position, semitandem, and tandem. For walking speed, participants were asked to walk at their usual pace over a 4-meter course. Participants could use an aid (eg, cane, walker, or other walking aid) if necessary but not the assistance of another person. For chair stands, participants were asked to stand up and sit down 5 times, as quickly as possible, from a straight-backed chair without using their arms. Each of the 3 performance measures was assigned a score ranging from 0 to 4, with 4 indicating the highest level of performance and 0 indicating an inability to complete the test. A summary score ranging from 0 (worst performers) to 12 (best performers) was calculated by adding balance, walking speed, and chair-stand scores (18,19).

Handgrip strength was measured using a hydraulic grip strength dynamometer (Jamar). Participants were seated with their elbow flexed at 90 degrees and performed 3 maximal contractions with a 60-second rest period between each contraction (20). The physical function subscale of the Medical Outcomes Short-Form 36-item questionnaire quantified physical functioning (21). The physical function subscale questionnaire asks about the ability to complete 10 tasks, including moderate- and vigorous-intensity activities, lifting groceries, climbing stairs, and walking various distances ranging from 1 block to more than 1 mile. The composite physical function subscale is scored by averaging the 10 responses, with each response ranging from 0 to 100 and higher values indicating better physical function. Sit-to-stand power (watts) and relative power (watts per kilogram of body weight) were calculated using established equations (22).

### Other measures

Demographic characteristics, including age and sex, were self-reported. Clinical characteristics, including cancer stage, planned chemotherapy duration, fluoropyrimidine type, and oxaliplatin use, were abstracted from a combination of pathology reports and physician records. Frailty was defined by a short physical performance battery score no more than 9 and dual-energy X-ray absorptiometry quantified appendicular lean mass less than 19.75 kg for males and less than 15.02 kg for females per the Sarcopenia and Physical fRailty IN older people: multi-componenT Treatment strategies (SPRINTT) consortium (23). Sarcopenia was defined as a handgrip strength less than 26 kg for males and less than 16 kg for females and appendicular lean mass adjusted for body mass index less than 0.789 for males and less than 0.512 for females per the Foundation for the National Institutes of Health Biomarkers Consortium Sarcopenia Project (FNIH) Sarcopenia Project (24). Physical limitation was defined as a composite short physical performance battery score of no more than 9 per the Lifestyle Interventions and Independence for Elders (LIFE) Study (25). Chemotherapy relative dose intensity was abstracted from the electronic medical record, and patient-reported chemotherapy toxicity was assessed using the Patient-Reported Outcomes version of the Common Terminology Criteria for Adverse Events, as previously described (10).

### Statistical analysis

The sample size was selected to provide statistical power to detect treatment effects for the coprimary endpoints of chemotherapy relative dose intensity and patient-reported chemotherapy-related toxicities (10). The minimally clinically significant difference for short physical performance battery score is 0.3-0.8 units (26). Based on prior studies in older adults without cancer (27,28), this analysis had 80% statistical power to detect a mean treatment difference of 0.6 units on the short physical performance battery score using a 2-sided *t* test with a 5% type-I error rate.

The statistical analysis included all participants randomly assigned under the intention-to-treat principle. The primary contrast quantified the effect of resistance training compared with usual care control. A logistic regression model quantified the association (odds ratio [OR]) between baseline factors and missing endpoint data at follow-up. Endpoints were analyzed using mixed models for repeated (pre, post) measures that included group, time (baseline, end of study), group-by-time interaction terms (to quantify the between-group difference over time), the baseline value of the dependent variable, and stratification factors used in the minimization allocation procedure. The adjusted between-group mean difference was quantified as the estimated treatment difference with corresponding 95% confidence intervals (CIs) and 2-sided *P* values. The 95% confidence intervals and *P* values were not adjusted for multiplicity and should not be used to infer definitive treatment effects. Interaction terms of group, time, and subgroup were included in regression models to quantify the heterogeneity of estimated treatment differences for exploratory hypothesis-generating subgroup analyses. The relationship between physical function and chemotherapy relative dose intensity and patient-reported chemotherapy-related toxicities was quantified with the Pearson correlation coefficient. Analyses were done using R (Version 4.1.0).

## Results

Between February 2018 and September 2021, a total of 181 participants were randomly assigned, with endpoint data collection ending in March 2022. Participants had a mean (SD) age of 55.2 (12.8) years (67 participants [37.0%] were 60 years and older, and 95 [52.5%] were male; Table 1). At baseline, 59 (35.1%) participants had frailty, 38 (23.3%) had sarcopenia, and 29 (16.0%) had a composite short physical performance battery score of no more than 9, indicative of suboptimal physical functioning. Participants randomly assigned to the resistance training group completed a median of 1.4 sessions (interquartile range [IQR] = 0.6-1.7 sessions) per week, using a weight that was 62% (IQR: 53-70%) of the estimated 1-repetition maximum of 3 sets (IQR: 2.6-3.6 sets) of 7.5 repetitions (IQR: 6.4-8.7 repetitions) [60.6% adherence (29)]. The average supplemental protein intake was 19.5 grams (12.7).

**Table 1.** Baseline characteristics overall and by randomly assigned group

Characteristic	Overall, No. (%)	Resistance training, No. (%)	Usual care control, No. (%)
	(n = 181)	(n = 90)	(n= 91)
Age, y			
Continuous, mean (SD)	55.2 (12.8)	56.3 (12.9)	54.2 (12.6)
Categorical			
Younger than 60	114 (63.0)	54 (60.0)	60 (65.9)
60 and older	67 (37.0)	36 (40.0)	31 (34.1)
Sex			
Female	86 (47.5)	44 (48.9)	42 (46.2)
Male	95 (52.5)	46 (51.1)	49 (53.8)
Planned chemotherapy duration			
3 months	57 (31.5)	29 (32.2)	28 (30.8)
6 months	124 (68.5)	61 (67.8)	63 (69.2)
Planned fluoropyrimidine type			
Capecitabine	77 (42.5)	41 (45.6)	36 (39.6)
5-fluorouracil	104 (57.5)	49 (54.4)	55 (60.4)
Time of random assignment			
Before chemotherapy	97 (53.6)	51 (56.7)	46 (50.5)
After chemotherapy	84 (46.4)	39 (43.3)	45 (49.5)
Frailty^a^			
No	109 (64.9)	50 (61.0)	59 (68.6)
Yes	59 (35.1)	32 (39.0)	27 (31.4)
Unknown or missing	13	8	5
Sarcopenia^b^			
No	125 (76.7)	60 (73.2)	65 (80.2)
Yes	38 (23.3)	22 (26.8)	16 (19.8)
Unknown or missing	18	8	10
Short physical performance battery score, no. (%)			
>9	152 (84.0)	75 (83.3)	77 (84.6)
≤9	29 (16.0)	15 (16.7)	14 (15.4)

a Frailty was defined by a short physical performance battery score of no more than 9 and appendicular lean mass less than 19.75 kg for males and less than 15.02 for females per the SPRINTT consortium (23). FNIH = Foundation for the National Institutes of Health Biomarkers Consortium Sarcopenia Project; SPRINTT = Sarcopenia and Physical fRailty IN older people: multi-componenT Treatment strategies.

b Sarcopenia was defined as a handgrip strength less than 26 kg for males and less than 16 kg for females and appendicular lean mass adjusted for body mass index less than 0.789 for males and less than 0.512 for females per the FNIH Sarcopenia Project (24).

**Figure 1.**
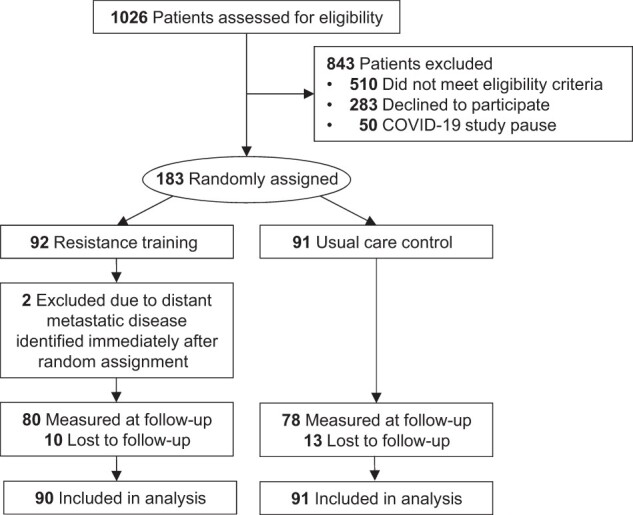
Flow of participants and ascertainment of physical function endpoints by randomly assigned group.

At chemotherapy completion, physical functioning endpoint data were provided by 158 (87.3%) participantsQ22 (Figure 1). Participants who did not provide physical functioning endpoint data were more likely to receive a planned chemotherapy duration of 6 months vs 3 months (OR = 5.5, 95% CI = 1.4 to 28.2); no other measured baseline factors, including random group assignment (OR = 0.75, 95% CI = 0.32 to 1.78), were associated with missing endpoint data.

### Short physical performance battery

Compared with usual care control, resistance training did not statistically significantly change the composite short physical performance battery score (estimated treatment difference = −0.01 points, 95% CI = −0.32 to 0.31 points; *P* = .98; Table 2), balance score (estimated treatment difference = 0.01 points, 95% CI = −0.10 to 0.11 points; *P* = .93), gait speed score (estimated treatment difference = 0.08 points, 95% CI = −0.06 to 0.22 points; *P* = .28), and sit-to-stand score (estimated treatment difference = −0.08 points, 95% CI = −0.29 to 0.13 points; *P* = .46). Compared with usual care control, resistance training did not statistically significantly change the 4-meter gait time (estimated treatment difference = −0.06 seconds, 95% CI = −0.32 to 0.21 seconds; *P* = .69) or sit-to-stand time (estimated treatment difference = 0.60 seconds, 95% CI = −0.13 to 1.35 seconds; *P* = .11; Table 3).

**Table 2.** Physical functioning outcomes at baseline and change during chemotherapy

Endpoint	Randomly assigned group	Baseline mean (SD)	Mean change (SE)^a^	Treatment difference (95% CI)^a^	*P*
Short physical performance battery, score, 0-12	Usual care control	10.8 (1.4)	0.0 (0.11)	0.00 (Referent)	
	Resistance training	11.0 (1.4)	0.0 (0.11)	−0.01 (−0.32 to 0.31)	.98
Balance, score, 0-4	Usual care control	3.9 (0.3)	−0.08 (0.04)	0.00 (Referent)	
	Resistance training	3.9 (0.3)	−0.07 (0.04)	0.01 (−0.10 to 0.11)	.93
Gait speed, score, 0-4	Usual care control	3.9 (0.4)	−0.05 (0.05)	0.0 (Referent)	
	Resistance training	3.8 (0.5)	0.02 (0.05)	0.08 (−0.06 to 0.22)	.28
Sit-to-stand, score, 0-4	Usual care control	3.0 (1.0)	0.12 (0.08)	0.00 (Referent)	
	Resistance training	3.2 (1.0)	0.04 (0.08)	−0.08 (−0.29 to 0.13)	.46
Handgrip strength, kg	Usual care control	38.0 (12.2)	−0.59 (0.92)	0.00 (Referent)	
	Resistance training	37.1 (14.1)	0.91 (0.93)	1.50 (−1.06 to 4.05)	.25
Physical function subscale Short Form–36, score, 0-100	Usual care control	77.9 (20.3)	−2.46 (2.34)	0.00 (Referent)	
	Resistance training	79.4 (20.6)	−6.01 (2.35)	−3.55 (−10.03 to 2.94)	.28

a Models are adjusted for baseline value of the dependent variable and random assignment stratification factors of sex (male vs female), cancer stage (II vs III), planned chemotherapy duration (3 months vs 6 months), planned chemotherapy regimen (FOLFOX vs CAPOX vs capecitabine monotherapy vs 5-fluorouracil and leucovorin), and study center (Kaiser Permanente Northern California vs Dana-Farber Cancer Institute vs Penn State Cancer Institute). CI = confidence interval.

**Table 3.** Exploratory physical functioning outcomes at baseline and change during chemotherapy

Endpoint	Randomized group	Baseline mean (SD)	Mean change (SE)^a^	Treatment difference (95% CI)^a^	*P*
4-m gait time, s	Usual care control	3.8 (1.0)	0.05 (0.10)	0.00 (Referent)	
	Resistance training	3.9 (1.1)	0.00 (0.10)	−0.06 (−0.32 to 0.21)	.69
Sit-to-stand, s	Usual care control	12.5 (4.0)	−0.70 (0.26)	0.00 (Referent)	
	Resistance training	11.5 (3.6)	−0.11 (0.26)	0.60 (−0.13 to 1.35)	.11
Sit-to-stand mean power, w	Usual care control	229.6 (112.5)	12.40 (7.25)	0.00 (Referent)	
	Resistance training	243.1 (110.7)	7.70 (7.21)	−4.70 (−24.7 to 15.33)	.65
Relative sit-to-stand mean power, w/kg	Usual care control	2.8 (1.1)	0.17 (0.08)	0.00 (Referent)	
	Resistance training	3.0 (1.0)	0.12 (0.08)	−0.05 (−0.29 to 0.18)	.67

a Models are adjusted for baseline value of the dependent variable and random assignment stratification factors of sex (male vs female), cancer stage (II vs III), planned chemotherapy duration (3 months vs 6 months), planned chemotherapy regimen (FOLFOX vs CAPOX vs capecitabine monotherapy vs 5-fluorouracil and leucovorin), and study center (Kaiser Permanente Northern California *vs* Dana-Farber Cancer Institute *vs* Penn State Cancer Institute). CI = confidence interval.

In exploratory subgroup analyses, compared with usual care control, resistance training increased the composite short physical performance battery score (estimated treatment difference = 0.97, 95% CI = 0.07 to 1.87; *P* = .04) among participants with a short physical performance battery score of no more than 9 at baseline (Supplementary Figure 1, available online). There were no meaningful subgroup effects for the balance score (Supplementary Figure 2, available online). Compared with usual care control, resistance training increased the gait-speed score in participants aged 60 years and older (estimated treatment difference = 0.26, 95% CI = 0.04 to 0.48), males (estimated treatment difference = 0.18, 95% CI = 0.03 to 0.32), and those randomly assigned after starting chemotherapy (estimated treatment difference = 0.17, 95% CI = 0.02 to 0.31; Supplementary Figure 3, available online). There were no meaningful subgroup effects for the sit-to-stand score (Supplementary Figure 4, available online). Subgroup effects were similar for 4-meter gait-speed time (Supplementary Figure 5, available online) and sit-to-stand time (Supplementary Figure 6, available online).

### Handgrip strength

Compared with usual care control, resistance training did not statistically significantly change isometric handgrip strength (estimated treatment difference = 1.50 kg, 95% CI = −1.06 to 4.05 kg; *P* = .25). In exploratory subgroup analyses, compared with usual care control, resistance training increased isometric handgrip strength in participants aged younger than 60 years (estimated treatment difference = 3.87 kg, 95% CI = 0.47 to 7.26 kg; Supplementary Figure 7, available online).

### Self-reported physical functioning

Compared with usual care control, resistance training did not statistically significantly change self-reported physical functioning (estimated treatment difference = −3.55 points, 95% CI = −10.03 to 2.94 points; *P* = .28). In exploratory subgroup analyses, compared with usual care control, resistance training decreased self-reported physical functioning in males (estimated treatment difference = −9.80, 95% CI = −18.88 to −0.73; Supplementary Figure 8, available online).

### Lower-extremity muscle power

Compared with usual care control, resistance training did not statistically significantly change absolute lower-extremity muscle power (estimated treatment difference = −4.70 watts, 95% CI = −24.7 to 15.33 watts; *P* = .65) or relative lower-extremity muscle power (estimated treatment difference = −0.05 watts per kilogram of body weight, 95% CI = −0.29 to 0.18 watts per kilogram of body weight; *P* = .67). In exploratory subgroup analyses, compared with usual care control, resistance training reduced lower-extremity muscle power (estimated treatment difference = −22.05 watts, 95% CI = −40.80 to −3.29 watts) and relative lower-extremity muscle power (estimated treatment difference = −0.26 watts per kilogram of body weight, 95% CI = −0.49 to −0.02 watts per kilogram of body weight) in females (Supplementary Figures 9 and 10, available online).

### Exploratory correlational analyses

**Figure 2.**
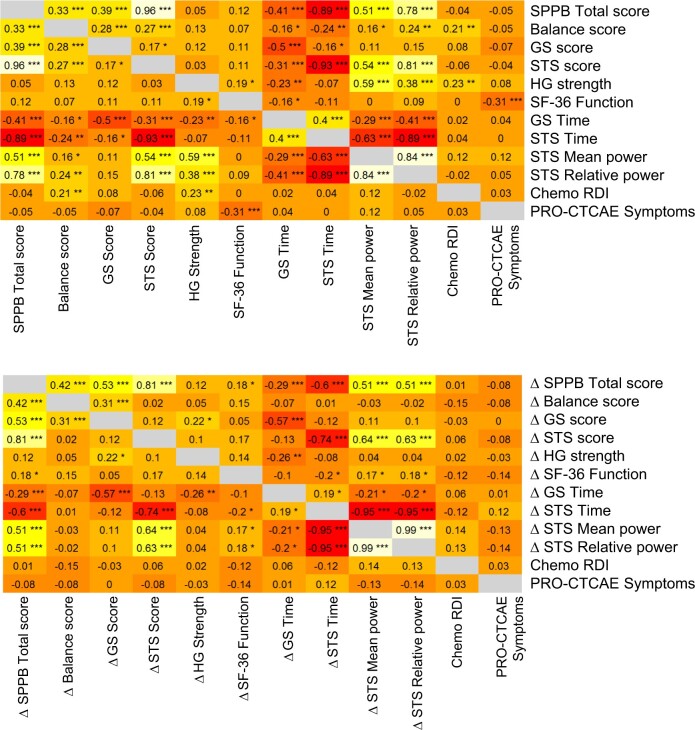
Correlational analyses of physical functioning outcomes at baseline (**top panel**) and change from baseline to end of chemotherapy (**bottom panel**) with chemotherapy relative dose intensity and the Patient-Reported Outcomes version of the Common Terminology Criteria for Adverse Events. *P < .05; **P < .01; ***P < .001. GS = gait speed; HG = handgrip; PRO-CTCAE = Patient-Reported Outcomes version of the Common Terminology Criteria for Adverse Events; SF-36 = Short Form–36; RDI = relative dose intensity; SPPB = short physical performance battery; STS = sit to stand.

At baseline, the short physical performance battery balance score (*r *=* *0.21, 95% CI = 0.07 to 0.35; *P* = .005) and handgrip strength (*r *=* *0.23, 95% CI = 0.09 to 0.36; *P* = .002) correlated with chemotherapy relative dose intensity and the Short Form–36 physical function subscale correlated with patient-reported chemotherapy toxicity (*r *=* *−0.31, 95% CI = −0.44 to −0.17; *P* < .001; Figure 2). Change in physical function was not associated with chemotherapy relative dose intensity or patient-reported chemotherapy toxicity.

## Discussion

In this multicenter trial of 181 colon cancer survivors, random assignment to home-based resistance training did not prevent physical function decline compared with usual care control during postoperative chemotherapy. Baseline measures of physical function, including the short physical performance battery balance score and handgrip strength, were correlated with chemotherapy relative dose intensity, and the Short Form–36 physical function subscale correlated with patient-reported chemotherapy toxicity; changes in physical function did not correlate with chemotherapy relative dose intensity or patient-reported chemotherapy toxicity. These findings do not support prescribing home-based resistance training to all colon cancer survivors to prevent physical function decline during chemotherapy.

This trial extends knowledge regarding the role of resistance training on physical function outcomes in cancer survivors. In the Predicting OptimaL Cancer RehabIlitation and Supportive care (POLARIS) consortium, an individual participant meta-analysis of 34 randomized trials, any type of exercise had a small but statistically significant effect on self-reported physical functioning (*z* score = 0.18, 95% CI = 0.13 to 0.23) (30). Furthermore, supervised exercise training statistically significantly improved self-reported physical functioning more than unsupervised exercise training (difference in *z* scores = 0.10, 95% CI = 0.01 to 0.20) (30). Our resistance training program used home-based exercise with adjustable dumbbells to attenuate barriers to trial enrollment and exercise adherence but did not change objectively measured or self-reported physical functioning measures. Future trials must balance the convenience of home-based exercise oncology interventions with the probability of improving physical function (31).

In our exploratory subgroup analyses, participants with a short physical performance battery score of no more than 9 at baseline who were randomly assigned to the resistance training group experienced a 0.97-point increase in the composite short physical performance battery score compared with the usual care control group. Although this was a small subgroup (n* *=* *29; 16% of the total cohort), it demonstrates that vulnerable participants may benefit from resistance training during chemotherapy. The 0.97-point improvement exceeds the minimally significant difference of 0.3-0.8 units that patients can readily notice (26). A 1-point improvement in the composite short physical performance battery score is associated with a 12% relative risk reduction in the hazard of death in cancer survivors (9). This subgroup is also supported by the POLARIS consortium, which concluded that cancer survivors with the lowest physical function experience the largest exercise-induced improvements in physical function (32). Enriching clinical trials with participants with physical limitations or frailty and sarcopenia has enabled the field of gerontology to establish the efficacy of physical activity in reducing the incidence of mobility disability in older adults (23,25). Similar clinical trial enrichment strategies may be valuable to exercise oncology.

Physical functioning often declines in the year after a cancer diagnosis. Among 228 colorectal cancer survivors, self-reported physical functioning declined by 16 points (on a scale from 0 to 100, with higher scores indicating better function) in the year following diagnosis compared with cancer-free control participants (1). Nearly 1 in 3 colon cancer survivors report a clinically relevant decline in physical function from diagnosis to 1 year postoperatively (2). Older adults may be more vulnerable to declines in physical function after a cancer diagnosis (1,2,4). However, our data were inconsistent with the above-described reports; we did not observe any meaningful declines in objectively measured or self-reported physical functioning in the control group. The explanations for this are uncertain but may relate to the selected patient population, who were younger and had relatively high physical functioning at baseline. This may have created a ceiling effect and limited our ability to detect differences by randomized group.

The findings from this randomized trial are relevant to the ACSM exercise guidelines for cancer survivors (14). Our data fill a major research gap identified in the ACSM guidelines, which recognize the lack of objective physical function measures in exercise oncology clinical trials. ACSM recognizes that supervised exercise may be more effective than unsupervised or home-based interventions. Our data do not support prescribing home-based resistance training to all colon cancer survivors to prevent physical function decline during chemotherapy. However, resistance training may confer other health benefits (14). The decision to prescribe resistance training during chemotherapy may require shared decision making.

There are several limitations to this trial. This trial was designed to examine the effects of resistance training for which it is challenging to blind study participants to treatment assignment. However, most of our study endpoints were objectively measured, thus reducing the degree of reporting bias. The study sample was not recruited based on having poor physical functioning at baseline, which may have limited our ability to detect treatment effects. In the United States, the average age for colon cancer diagnosis is 69 years; however, the participants in this study were aged 55 years. Consequently, the prevalence of age-related syndromes, such as sarcopenia and frailty, was low and may have impeded our ability to detect treatment effects. This study only investigated resistance training; therefore, we cannot comment on the effects of aerobic training. The average adherence to the resistance training program was 60.6%; it is uncertain if improved adherence would have altered our conclusions. Outcome measures were not repeatedly measured within timepoint; thus, we cannot rule out measurement error.

This trial has several strengths. The randomized design and the use of objective and self-reported physical function outcome measures allowed for efficient and comprehensive estimation of treatment effects. Participants retention was outstanding (87.2%) despite the concurrent SARS-CoV-2 pandemic. Ascertainment of outcome measures did not differ by the randomly assigned group, and baseline physical functioning was not associated with loss to follow-up.

In conclusion, among colon cancer survivors with relatively high physical functioning at baseline receiving postoperative chemotherapy, random assignment to home-based resistance training did not prevent physical function decline compared with usual care control. Additional well-designed randomized trials are required to determine if exercise benefits patients vulnerable to declines in physical function during chemotherapy, such as older adults and those with low physical functioning or sarcopenia and frailty.

## Supplementary Material

pkae058_Supplementary_Data

## Data Availability

Participants did not consent to public sharing of individual-level data.
