# Supplementary material for: Effect of resistance training on physical function during chemotherapy in colon cancer
Source: JNCI Cancer Spectr. 2024 Jul 16;8(4):pkae058. doi: 10.1093/jncics/pkae058 (PMC11285783; doi:10.1093/jncics/pkae058)
Supplement: pkae058_Supplementary_Data [file pkae058_supplementary_data.docx]

**Effect of Resistance Training on Physical Function During Chemotherapy in Colon Cancer**

Justin C. Brown, Ph.D.,^1,2,3^ Shengping Yang, Ph.D.,^1^ Stephanie L.E. Compton, Ph.D.,^1^ Kristin L. Campbell, Ph.D.,^4^ Elizabeth M. Cespedes Feliciano, Sc.D.,^5^ Sara Quinney, Ph.D.,^6^ Barbara Sternfeld, Ph.D.,^5^ Bette J. Caan, Dr.P.H.,^6^ Jeffrey A. Meyerhardt, M.D., M.P.H.,^7^ Kathryn H. Schmitz, Ph.D.^8^

^1^Pennington Biomedical Research Center, 6400 Perkins Rd, Baton Rouge, LA 70808, USA; ^2^LSU Health Sciences Center New Orleans School of Medicine, 1901 Perdido St, New Orleans, LA 70112, USA; ^3^Stanley S. Scott Cancer Center, Louisiana State University Health Sciences Center, 533 Bolivar St, New Orleans, LA, 70112, USA; ^4^University of British Columbia, Vancouver, 2177 Westbrook Mall, British Columbia, V6T 1Z1, Canada; ^5^Kaiser Permanente Northern California, 2000 Broadway, Oakland, CA 94612, USA; ^6^Indiana University School of Medicine, 950 W Walnut St, Indianapolis, IN, 46202, USA; ^7^Dana-Farber Cancer Institute, 450 Brookline Ave, Boston, MA 02215, USA; ^8^University of Pittsburgh, 5150 Centre Ave, Pittsburgh, PA 15232, USA

**Corresponding Author:**

Justin C. Brown, Ph.D.

6400 Perkins Road

Baton Rouge, LA 70808

Phone: 225-763-2715

Email: [Justin.Brown@pbrc.edu](mailto:Justin.Brown@pbrc.edu)

ORCiD: 0000-0001-7540-4913

**Short Title:** Resistance Training on Physical Function in Colon Cancer

**Supplementary Figure 1.** Subgroup analyses of the effect of resistance training versus usual care control on the Short Physical Performance Battery (SPPB) composite (score, 0−12)


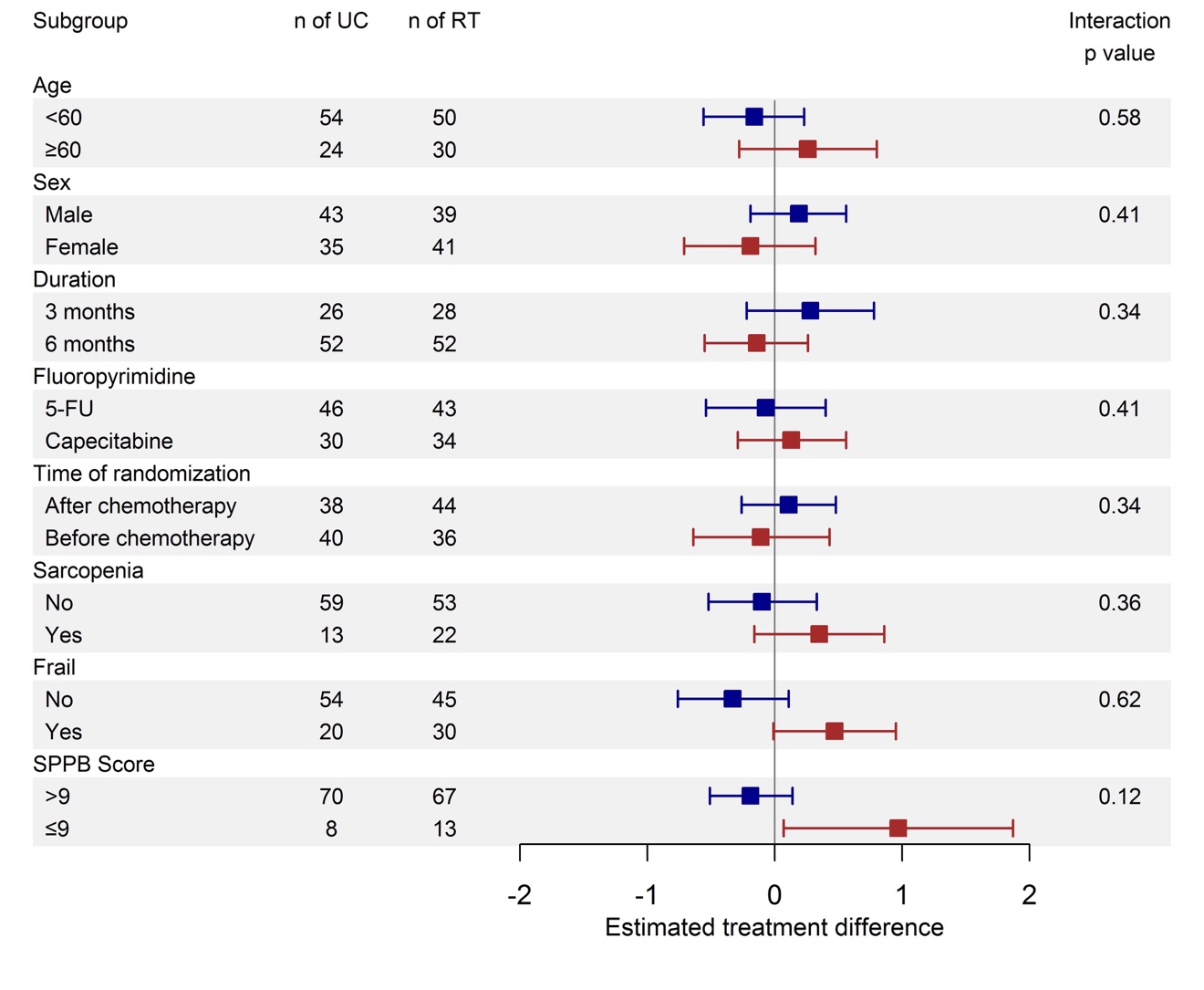


**Supplementary Figure 2.** Subgroup analyses of the effect of resistance training versus usual care control on the Short Physical Performance Battery (SPPB) balance (score, 0−4)

**
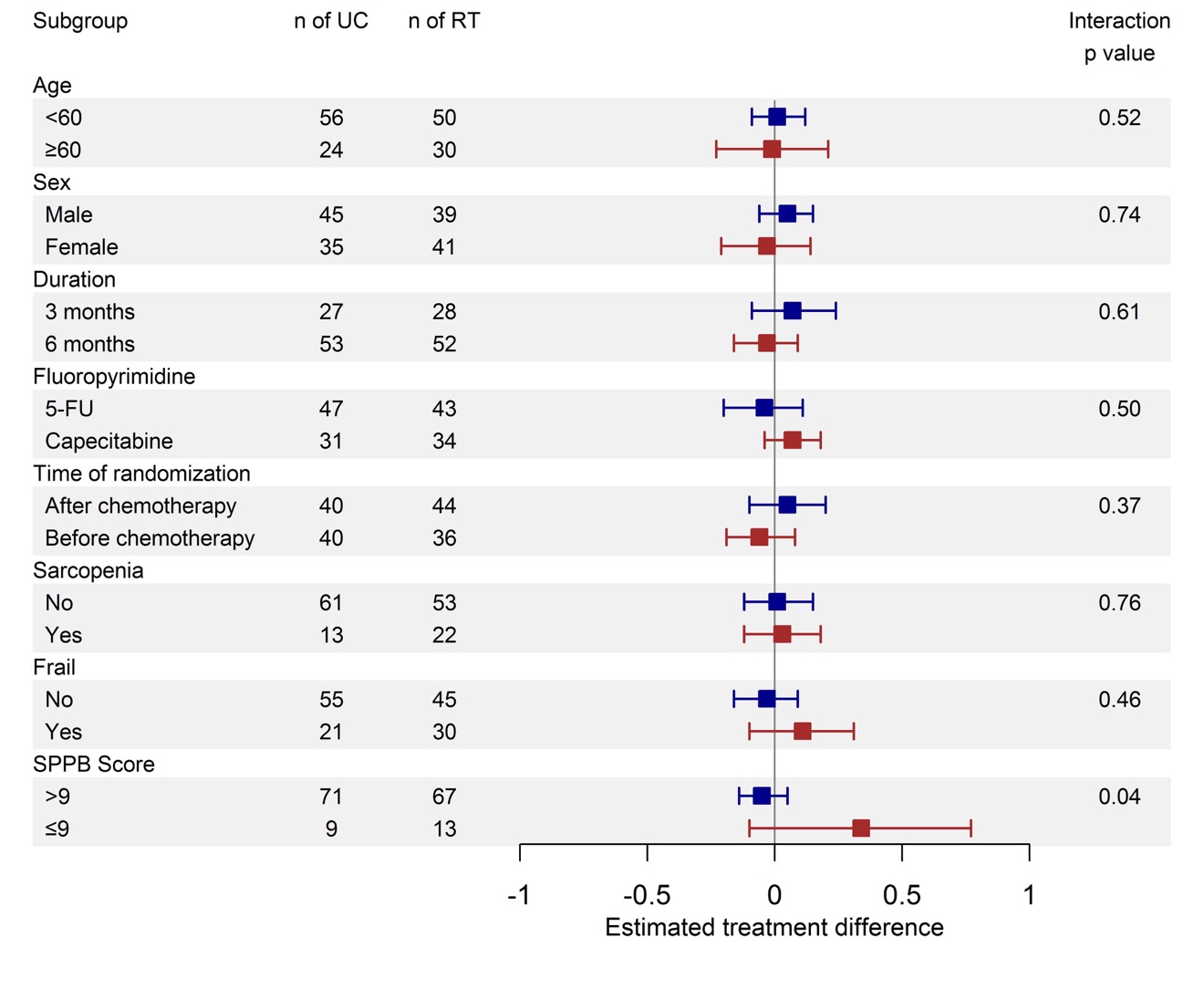
**

**Supplementary Figure 3.** Subgroup analyses of the effect of resistance training versus usual care control on the Short Physical Performance Battery (SPPB) gait speed (score, 0−4)

**
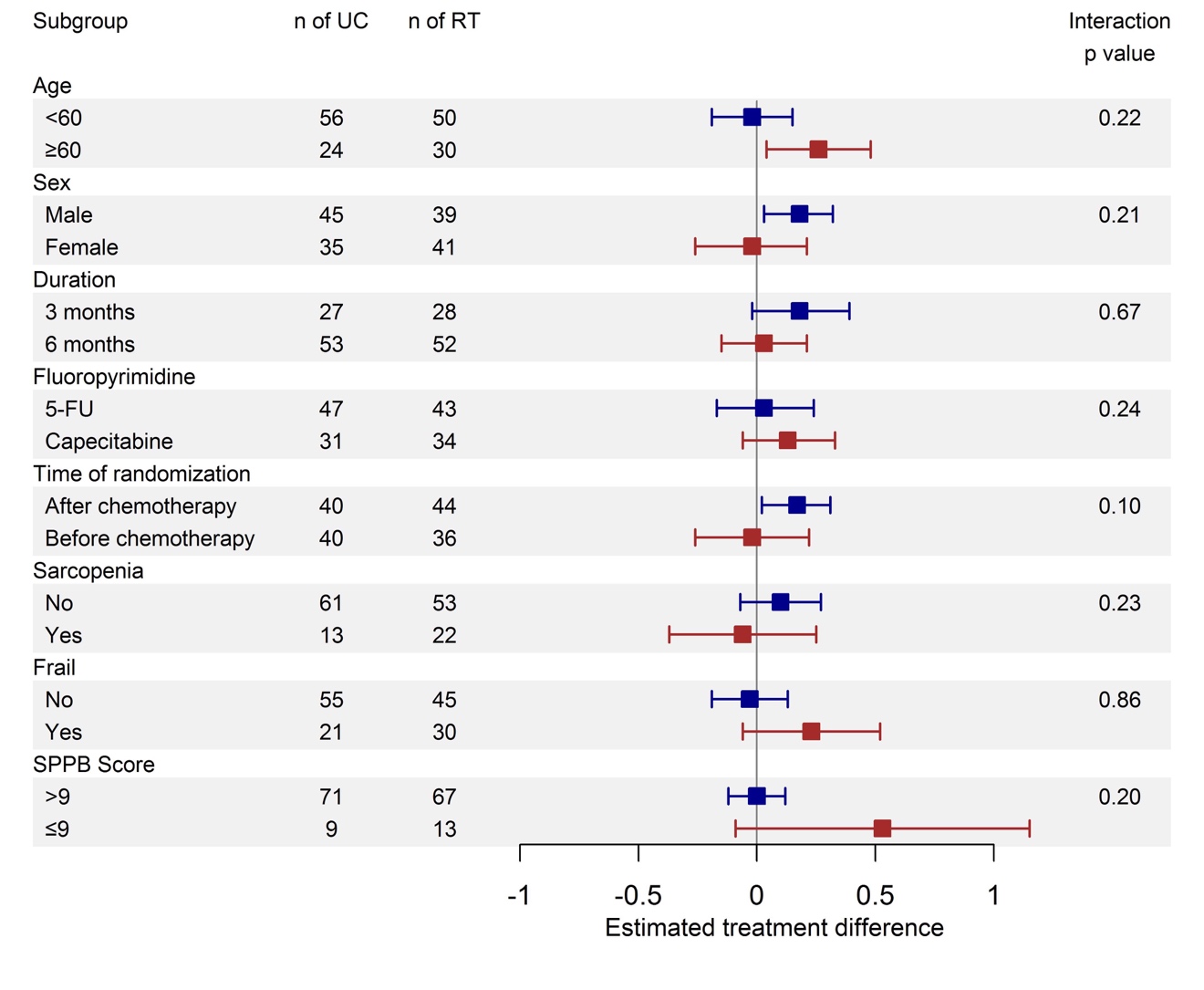
**

**Supplementary Figure 4.** Subgroup analyses of the effect of resistance training versus usual care control on the Short Physical Performance Battery (SPPB) sit-to-stand (score, 0−4)

**
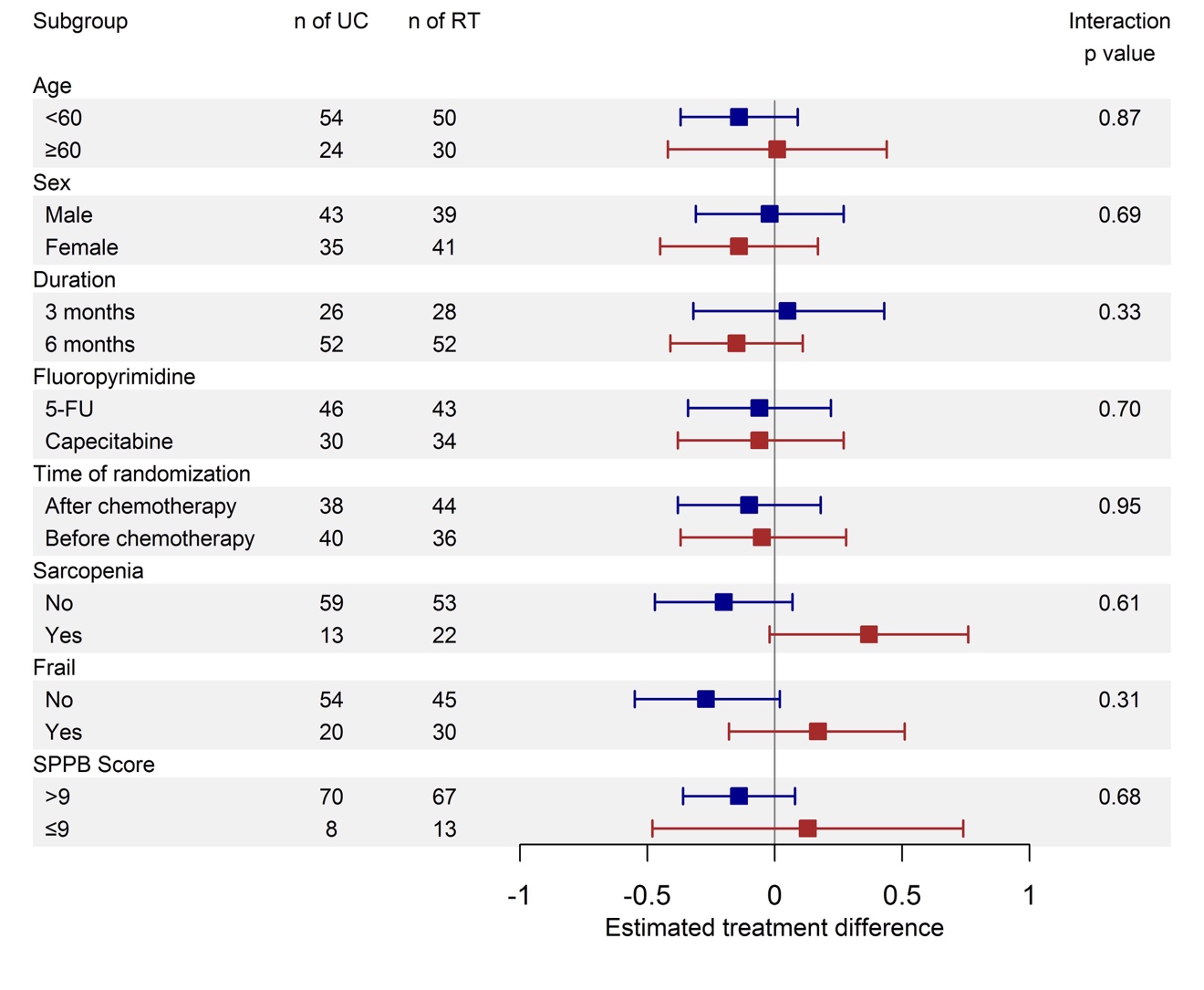
**

**Supplementary Figure 5.** Subgroup analyses of the effect of resistance training versus usual care control on the 4-meter gait time (seconds)

**
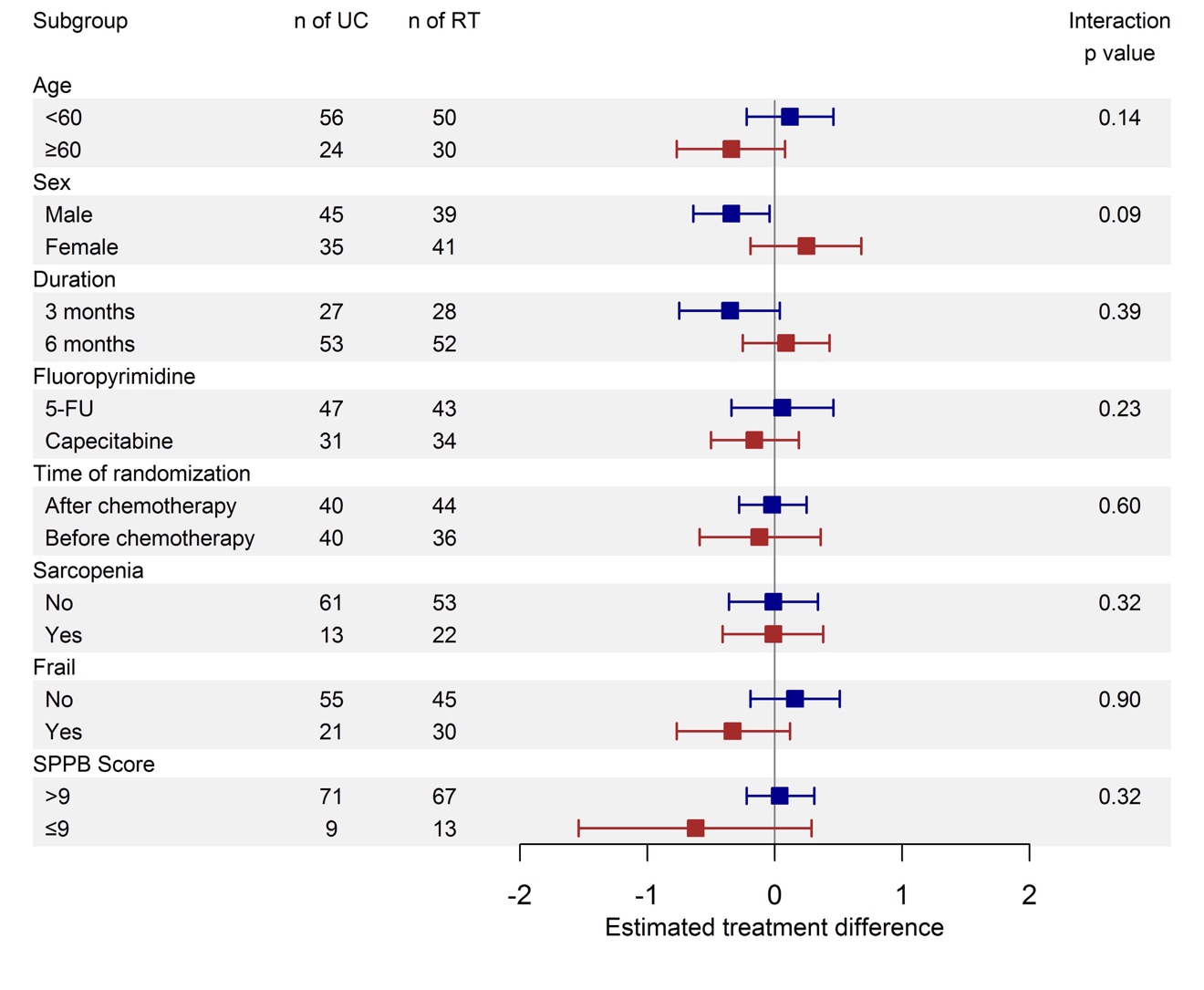
**

**Supplementary Figure 6.** Subgroup analyses of the effect of resistance training versus usual care control on the sit-to-stand time (seconds)

**
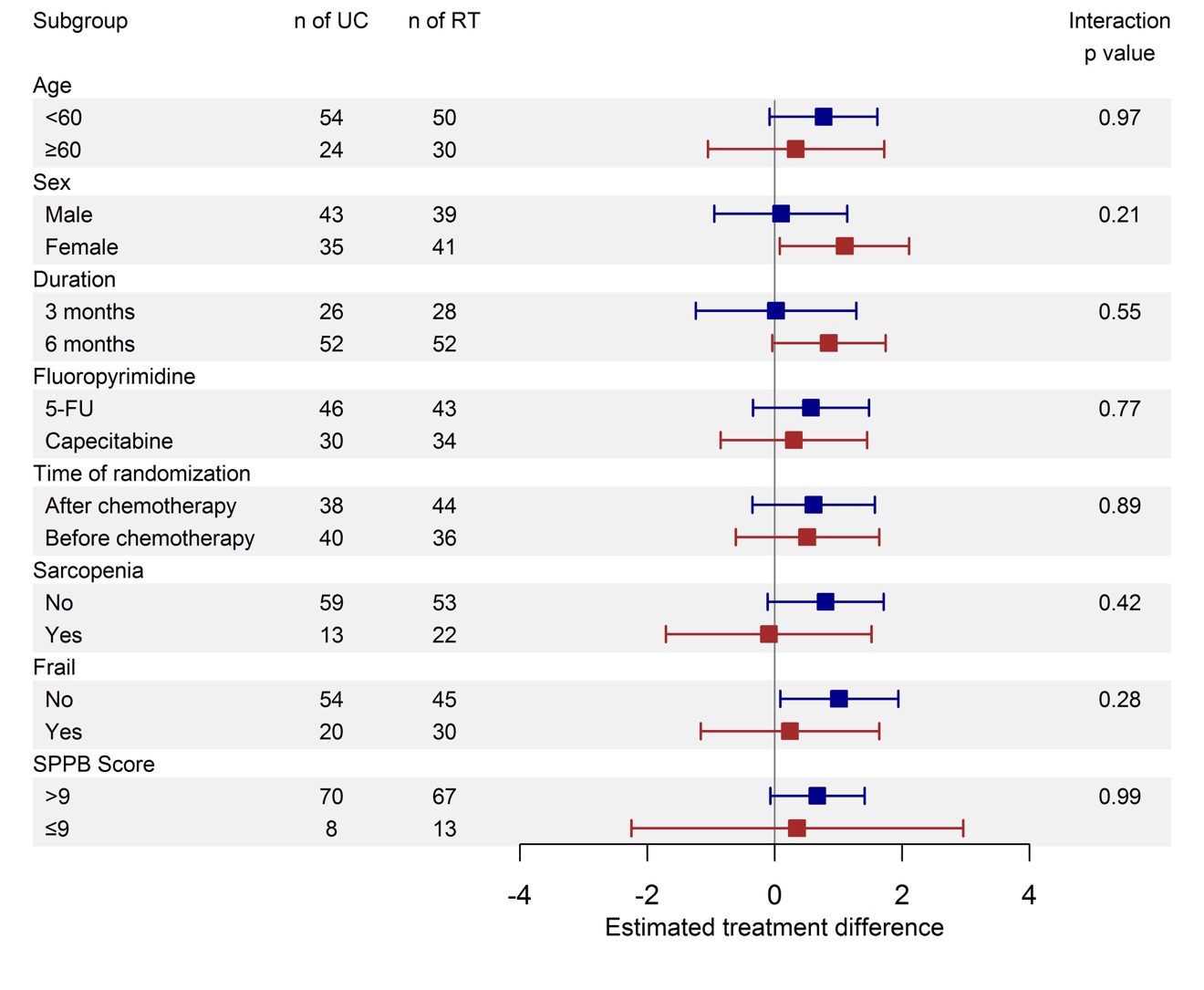
**

**Supplementary Figure 7.** Subgroup analyses of the effect of resistance training versus usual care control on handgrip strength (kilograms)

**
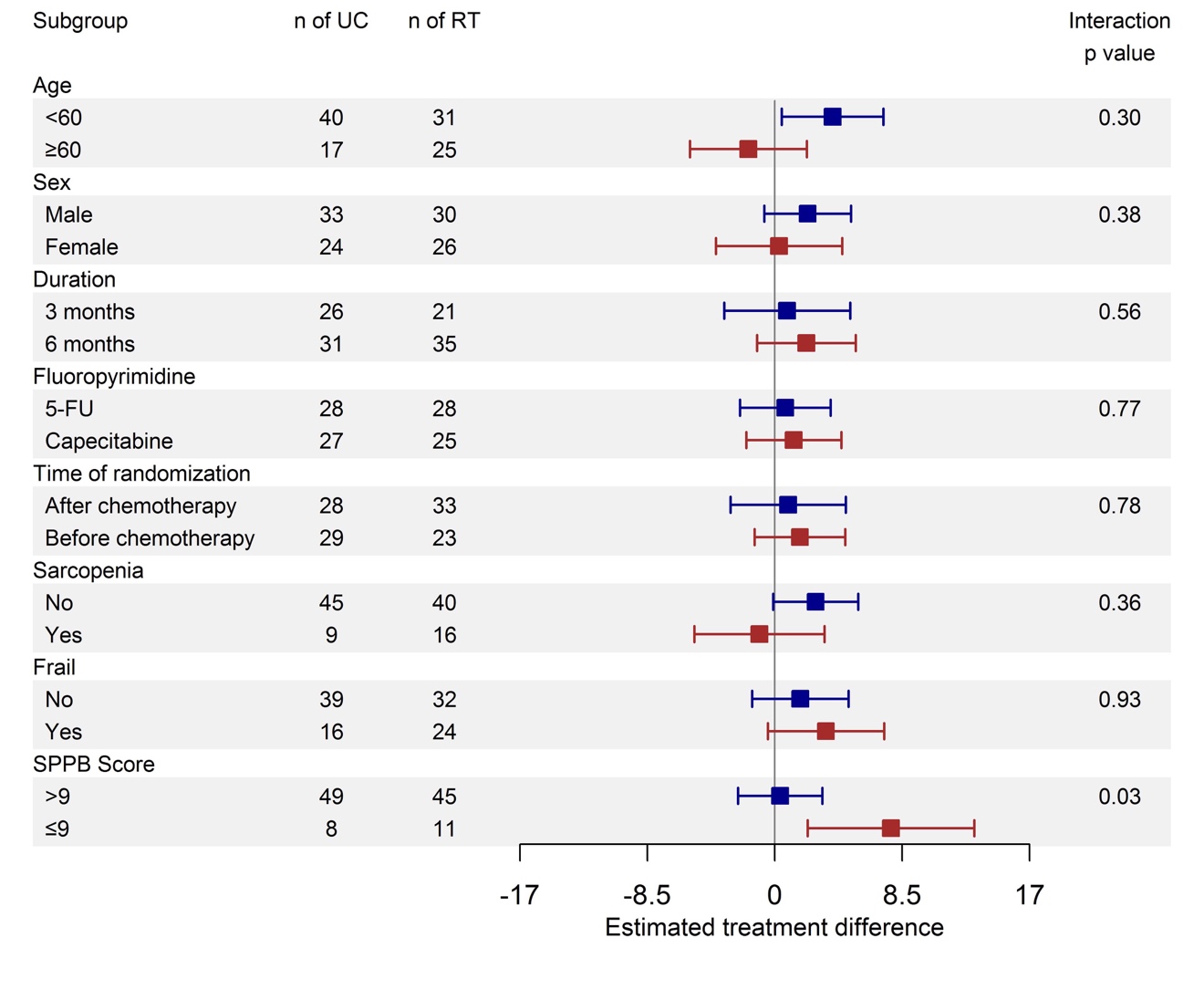
**

**Supplementary Figure 8.** Subgroup analyses of the effect of resistance training versus usual care control on the physical function subscale of the SF-36 survey score (score, 0−100)

**
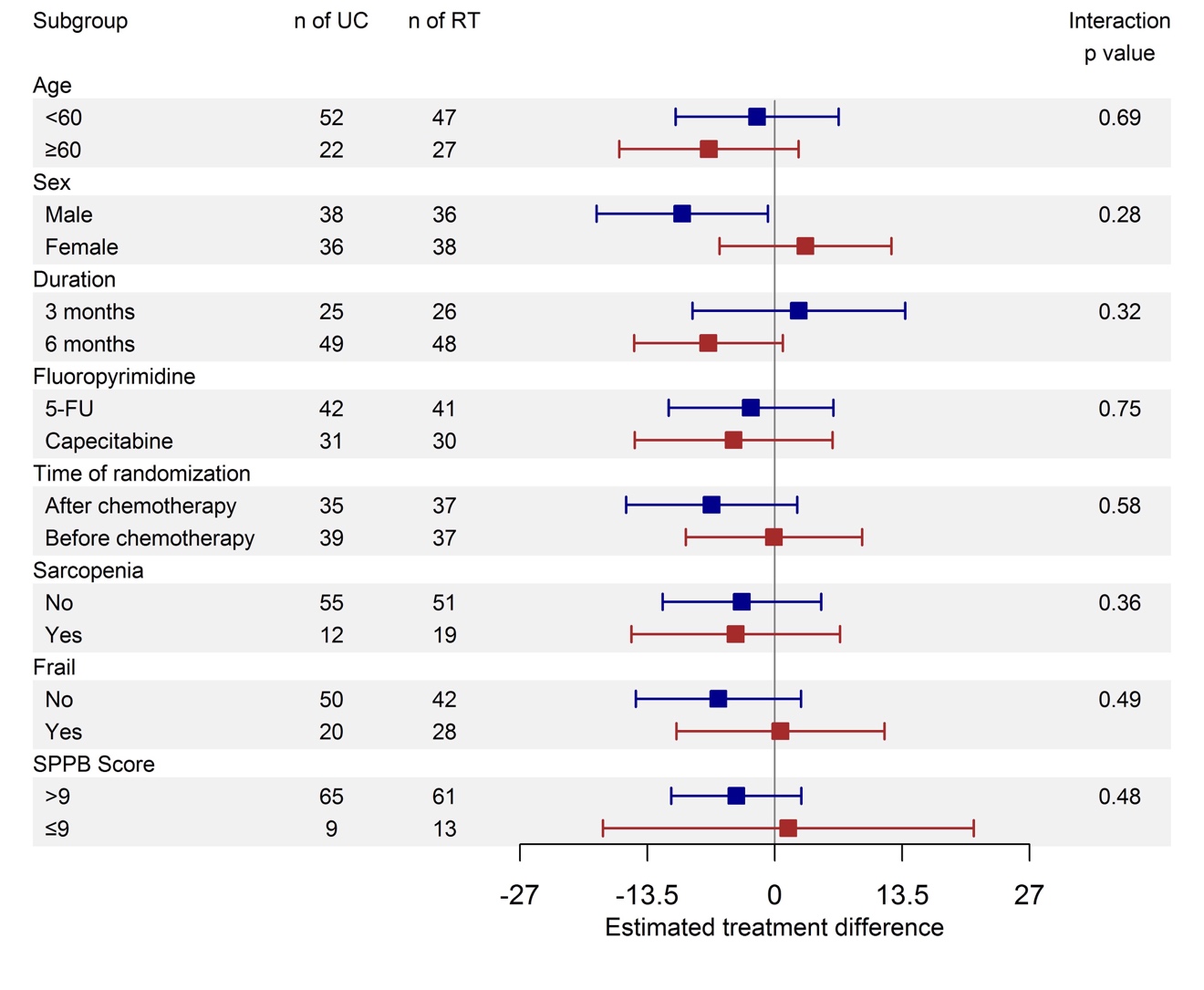
**

**Supplementary Figure 9.** Subgroup analyses of the effect of resistance training versus usual care control on the sit-to-stand mean power (watts)

**
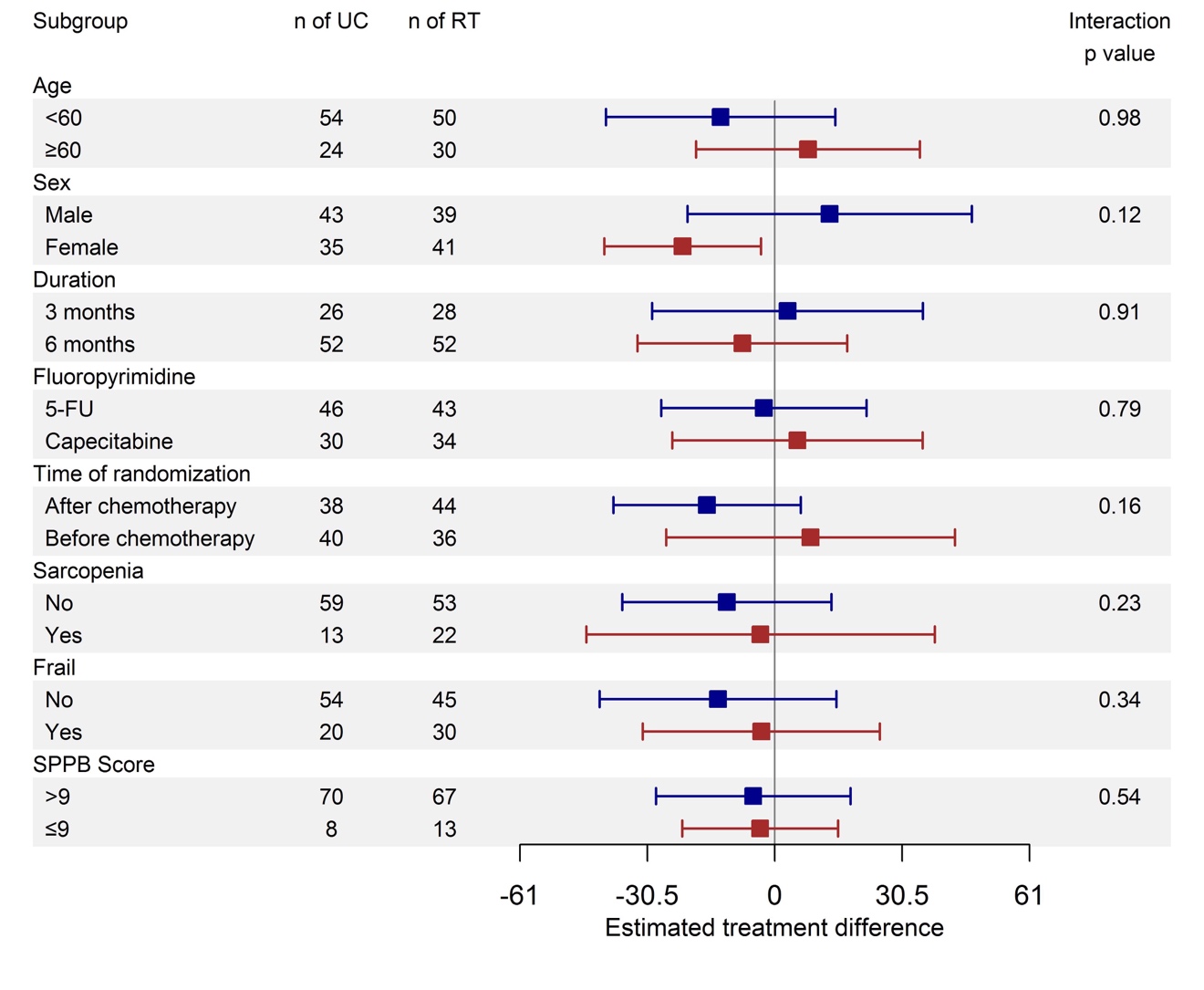
**

**Supplementary Figure 10.** Subgroup analyses of the effect of resistance training versus usual care control on the relative sit-to-stand mean power (watts per kilogram of bodyweight)

**
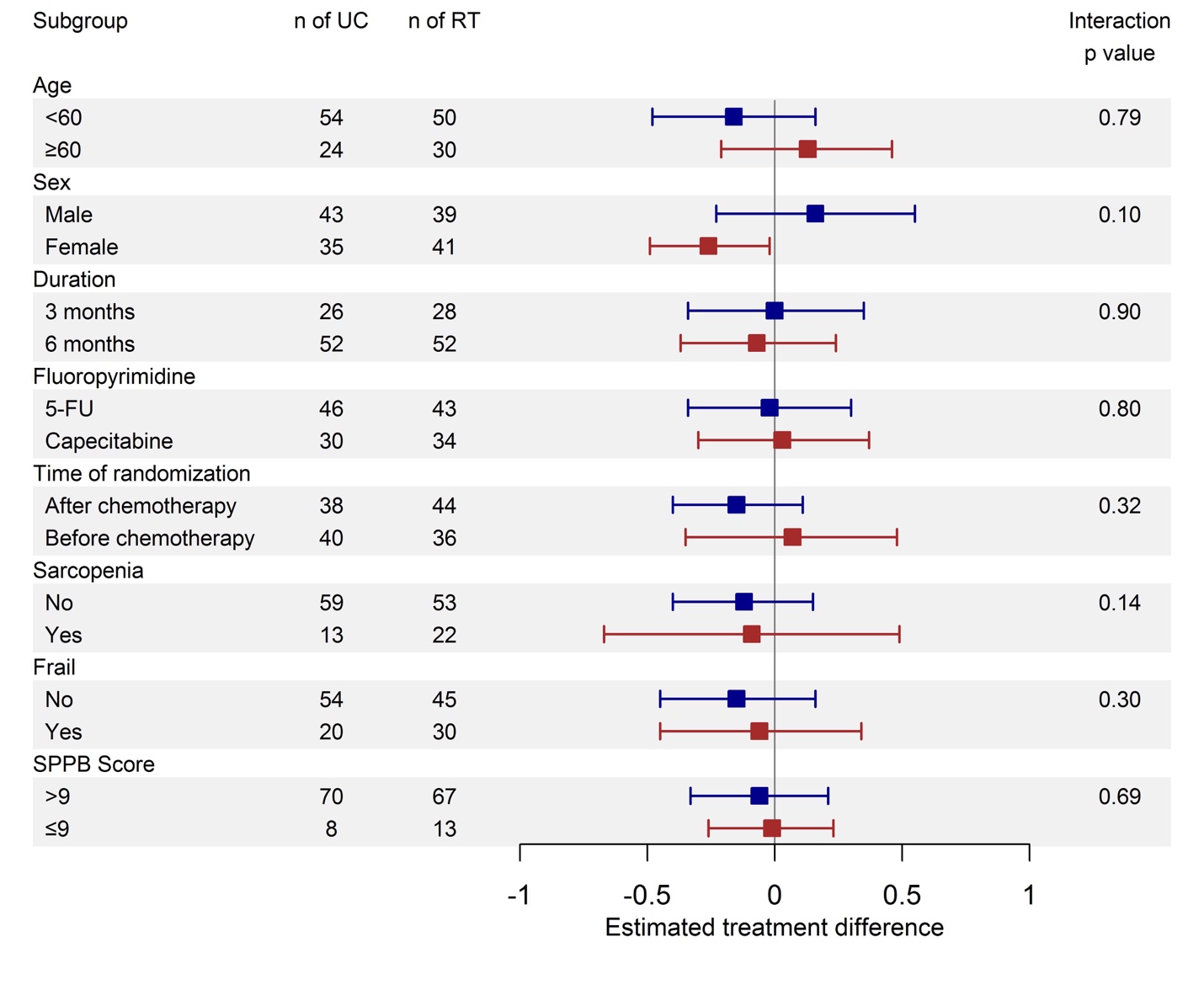
**
